# Anti-HERV-W_Env_ antibodies are correlated with seroreactivity against *Mycobacterium avium* subsp. *paratuberculosis* in children and youths at T1D risk

**DOI:** 10.1038/s41598-019-42788-5

**Published:** 2019-04-18

**Authors:** Magdalena Niegowska, Małgorzata Wajda-Cuszlag, Grażyna Stępień-Ptak, Joanna Trojanek, Jacek Michałkiewicz, Mieczysław Szalecki, Leonardo A. Sechi

**Affiliations:** 1 Laboratory of Microbiology, Department of Biomedical Sciences, University of Sassari, Sassari, Italy; 2 Clinic of Endocrinology and Diabetology, Children’s Memorial Health Institute, Warsaw, Poland; 3 Department of Clinical Microbiology and Immunology, Children’s Memorial Health Institute, Warsaw, Poland; 4 Department of Immunology, Collegium Medicum in Bydgoszcz, Nicolaus Copernicus University in Torun, Bydgoszcz, Poland; 5 Department of Medicine and Health Sciences, Jan Kochanowski University, Kielce, Poland

**Keywords:** Viral infection, Pre-diabetes

## Abstract

Recent evidence points at the role that human endogenous retroviruses (HERVs) may play through the activation of genes integrated across the human genome. Although a variety of genetic/epigenetic mechanisms maintain most HERVs silenced, independent environmental stimuli including infections may transactivate endogenous elements favoring pathogenic conditions. Several studies associated exposures to *Mycobacterium avium* subsp. *paratuberculosis* (MAP) with increased anti-MAP seroreactivity in T1D patients. Here, we assessed humoral responses against HERV envelope antigens (HERV-K_Env_ and HERV-W_Env_) and four MAP-derived peptides with human homologs in distinct populations: Sardinian children at T1D risk (rT1D) (n = 14), rT1D from mainland Italy (n = 54) and Polish youths with T1D (n = 74) or obesity unrelated to autoimmunity (OB) (n = 26). Unlike Sardinian rT1D, youths displayed increased anti-HERV-W_Env_ Abs prevalence compared to age-matched OB or healthy controls (24.32 vs. 11.54%, *p* = 0.02 for Polish T1D/OB and 31.48 vs. 11.90%, *p* = 0.0025 for Italian rT1D). Anti-HERV-K_Env_ responses showed variable trends across groups. A strong correlation between Abs levels against HERV-W_Env_ and homologous peptides was mirrored by time-related Abs patterns. Elevated values registered for HERV-W_Env_ overlaped with or preceded the detection of T1D diagnostic autoantibodies. These results support the hypothesis of MAP infection leading to HERV-W antigen expression and enhancing the production of autoantibodies in T1D.

## Introduction

Genetic heterogeneity characterizing type 1 diabetes (T1D) enhanced in the recent years the research on environmental components considered as putatively decisive factors underlying development of autoimmunity. However, despite numerous variables including diet, toxins, gut dysbiosis and viral infections assessed to date, no causal relationship with disease progression has been established. Complex and unclear T1D etiology suggests the involvement of various contributors acting on specific endpoints in a series of events. A large body of evidence points at the role that human endogenous retroviruses (HERVs) may play through the activation of genes integrated in multiple copies across the human genome as traces of ancestral infections^1^. The envelope protein of HERV-W and HERV-K families has been detected in patients with several immune-mediated and neurodegenerative diseases^2–4^ and reported to function as a superantigen due to the ability of inducing strong T cell responses^5^. The specific role of HERV envelope proteins in immune and neurological disorders, including T1D, has been recently reviewed^6^. In T1D, the initially described association with HERV-K has been questioned based on the ubiquitous nature of its antigens^7–9^, whereas HERV-W remains a valid object of investigation. A high expression of HERV-W envelope protein (HERV-W_Env_) observed in serum, peripheral blood mononuclear cells and pancreas of T1D patients has been recently correlated with macrophage infiltrates, inhibited insulin secretion in human Langerhans islets *in vitro* and corroborated by studies in transgenic mice^10^ which also develop autoantibodies against recombinant viral gp70 envelope protein increasing in titer along with disease progression^11^. GNbAC1 monoclonal antibody specifically targeting HERV-W_Env_ is currently under a phase-IIa clinical trial testing as a possible HERV-based therapeutic approach in T1D^12^. Although a variety of genetic and epigenetic mechanisms maintain most HERVs silenced, independent environmental stimuli such as infections may transactivate endogenous elements favoring pathogenic conditions^13^. Previously, we have described a significantly elevated seroreactivity of T1D subjects towards antigens sharing amino acid sequence homology derived from *Mycobacterium avium* subsp. *paratuberculosis* (MAP) and human proteins involved in the pathogenesis of diabetes: zinc transporter 8 (ZnT8) and proinsulin (PI)^14,15^. These epitopes were recognized by over 65% of T1D patients from independent cohorts in contrast to non-diabetic controls and patients affected by type 2 diabetes (T2D) who displayed specific responses to a very limited extent^16^. In the present study, we show a strong correlation between antibodies (Abs) directed against the assessed peptides and HERV-W_Env_ fragment in three populations at risk of T1D (rT1D) or with developed disease differing by age and biogeographic background. The observed time-related Abs patterns hint at a possible reactivation of HERV-W_Env_ following exposure to MAP that, through imbalance facilitated by cross-reacting homologous epitopes, may lead to the loss of immune tolerance.

## Results

### Seroreactivity against HERV_Env_ antigens in correlation with T1D clinical phase

**Figure 1:**
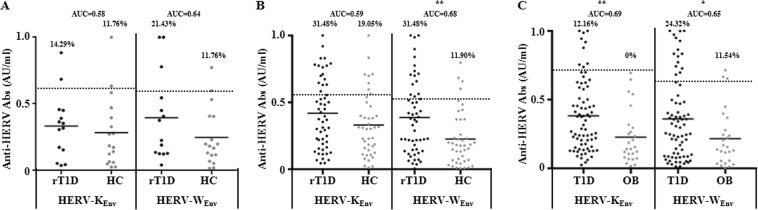
Prevalence of Abs against HERV_Env_ antigens in Sardinian rT1D children (**A**), rT1D youths from mainland Italy (**B**) and Polish T1D youths (**C**) along with the respective control groups. The dotted lines represent positivity thresholds calculated by ROC analysis. Dashed lines indicate the respective mean AU/ml values. Area under the curve (AUC) and statistical significance, when attained, are reported for each distribution.

In all analyzed groups, subjects at risk of or affected by T1D displayed higher Abs values against HERV_Env_ epitopes compared to HC and OB patients (Fig. 1). More pronounced differences of seroreactivity against the two peptides were registered among the youngest participants from Sardinia, for which the prevalence of anti-HERV-K_Env_ Abs reached similar values between children at T1D risk or HC (14.29 vs. 11.76%, respectively) and corresponded to elevated values for HERV-W_Env_ among patients followed up for autoimmunity, with unchanged levels in HC (21.43 vs. 11.76%, respectively).

Youths from mainland Italy showed differing trends. Elevated responses to both HERV-W_Env_ and HERV-K_Env_ were observed in over 31% of rT1D subjects. In contrast, HC presented significantly lower levels of anti-HERV-W_Env_ Abs (11.9%, *p* = 0.0025) with somewhat higher reactivity towards HERV-K_Env_ (19.05%).

Less pronounced, although highly significant reactivity against HERV_Env_ antigens were registered among Polish T1D patients whose prevalence of anti-HERV-K_Env_ Abs accounted for 12.16%, whilst no positive subject was found in the OB group (*p* = 0.0034). HERV-W_Env_ triggered more elevated responses in both clinical conditions, however marked difference was still maintained (24.32% for T1D vs. 11.54% for OB, *p* = 0.02).

### Correlation between reactivity against HERV_Env_ and homologous MAP/human epitopes

Previous screening for Abs against fragments of mycobacterial cation transporter MAP3865c_133–141_ and MAP3865c_125–133_, a hypothetical protein MAP2404c_70–85_, and a glucan branching protein MAP1,4*α*gbp_157–173_ alongside their respective human homologs ZnT8_186–194_, ZnT8_178–186_, PI_46–61_ and PI_64–80_ revealed elevated responsiveness of T1D and rT1D patients to the selected antigens when compared to reference controls^17,18^.

Even though Abs prevalence followed different patterns in the three assessed populations, values registered for either HERV-W_Env_ or HERV-K_Env_ correlated with MAP/PI epitopes regardless of the location and disease phase (Table 1). Particularly elevated R^2^ values were reached between HERV-W_Env_ and PI antigens in patients with established T1D. No correlation was found with single-point levels of diagnostic biomarkers or age groups within single cohorts, however the coefficient of determination increased proportionally considering the mean age of entire populations (children < younger youths (Italian) < older youths (Polish)). Less linear trends and slightly lower R^2^ were registered for HERV-K_Env_ among patients and for both antigens in youth control groups. In Sardinian healthy subjects, comparison between anti-HERV-K_Env_ and anti-MAP (PI homologs) Abs corresponded to higher coefficients than in relative rT1D children. Abs targeting MAP/ZnT8 homologous epitopes correlated overall to a weaker extent with each HERV_Env_ epitope but maintained high coefficient values for HERV-W_Env_ in Polish T1D group. As well, anti-ZnT8_186–194_ Abs corresponded to a marked correlation with reactivity towards HERV-W_Env_ in both populations of subjects at T1D risk.

**Table 1:** Relationship between HERV_Env_ antigens and homologous epitope pairs derived from MAP and human proteins expressed as R^2^. Values were obtained based on all available samples for single populations. For each peptide, R^2^ corresponding to patients are reported with underneath coeffcients relative to the respective control groups.

MAP/human antigen	HERV-K_Env_			HERV-W_Env_		
	Sardinian	Italian	Polish	Sardinian	Italian	Polish
MAP1,4*α*gbp_157–173_	0.7167	0.7154	0.6581	0.7008	0.8066	0.8149
	0.8489	0.6457	0.5271	0.7716	0.6535	0.5569
PI_64–80_	0.7822	0.7130	0.7630	0.7547	0.8151	0.8617
	0.4783	0.6725	0.5191	0.1507	0.6405	0.5871
MAP2404c_70–85_	0.7950	0.7155	0.7446	0.7984	0.7747	0.7963
	0.8603	0.5363	0.7590	0.6219	0.5187	0.7718
PI_46–61_	0.7816	0.7123	0.7229	0.7768	0.7974	0.8209
	0.7709	0.6009	0.7219	0.6709	0.5264	0.7560
MAP3865c_133–141_	0.6676	0.6586	0.6303	0.5790	0.6929	0.7102
	0.3907	0.5207	0.5352	0.1459	0.3980	0.5887
ZnT8_186–194_	0.7847	0.6506	0.6457	0.7017	0.7530	0.7186
	0.6430	0.6354	0.5996	0.5538	0.5591	0.6708
MAP3865c_125–133_	0.8335	0.6271	0.6746	0.8003	0.6815	0.7573
	0.3677	0.4851	0.4913	0.5303	0.4646	0.6471
ZnT8_178–186_	0.7616	0.5817	0.6825	0.7984	0.6194	0.8153
	0.7146	0.6213	0.4032	0.4333	0.5149	0.4742

### Temporal coincidence of Abs patterns

**Figure 2:**
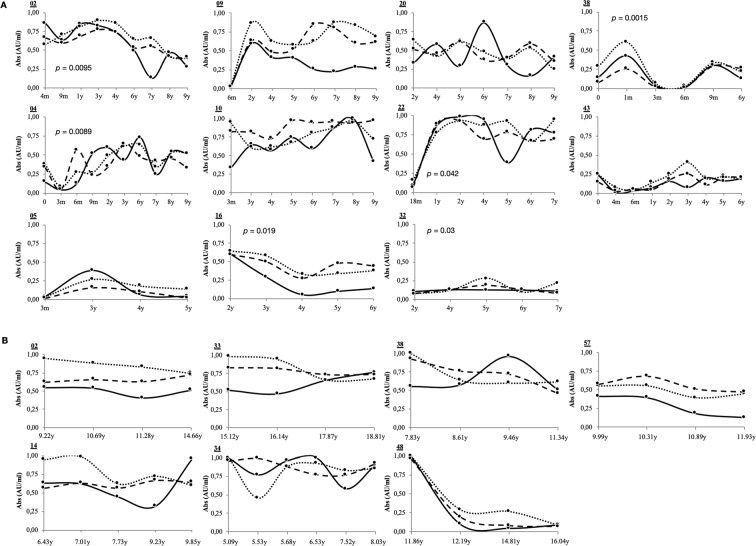
Time-related seroreactivity against HERV-W_Env_ and homologous MAP/human antigens in subjects at T1D risk from Sardinia (**A**) and mainland Italy (**B**). For graphical simplification, data relative to MAP/ZnT8 or MAP/PI sets are represented as separated means since values obtained for each of the four peptides included in their specific set fell into similar ranges. Results shown correspond to subjects for which at least 4 follow-up samples were available with patients’ identifiers reported above the Y axis of each graph. HERV-W_Env_: continuous line. MAP/PI: dashed line. MAP/ZnT8: dotted line. m: months. y: years.

Upon time-point analysis of Abs status in subjects for which follow-up samples were available (n = 13 for Sardinian children and n = 26 for Italian youths at T1D risk), the obtained values were classified in two sets based on peptide sequence homology: MAP/ZnT8 and MAP/PI (Fig. 2). Further comparison with Abs against HERV-W_Env_ confirmed the correlation in a time-related manner. Interestingly, value reads for HERV-W_Env_ were prone to fluctuations mirrored by variable Abs status against MAP/human epitopes during long observation periods. Moreover, they tended to raise or decrease in concert with or subsequently to variations of Abs specific for the homologous peptides assessed. Statistically significant results were obtained in 6 out of 11 Sardinian patients but not in the Italian rT1D group which may be due a lower number of time-point samples available. This may result from shifted fluctuations during which the maximal peaks of anti-HERV-W_Env_ Abs not necessarily correspond to minimal responses against MAP/human homologous antigens.

**Figure 3:**
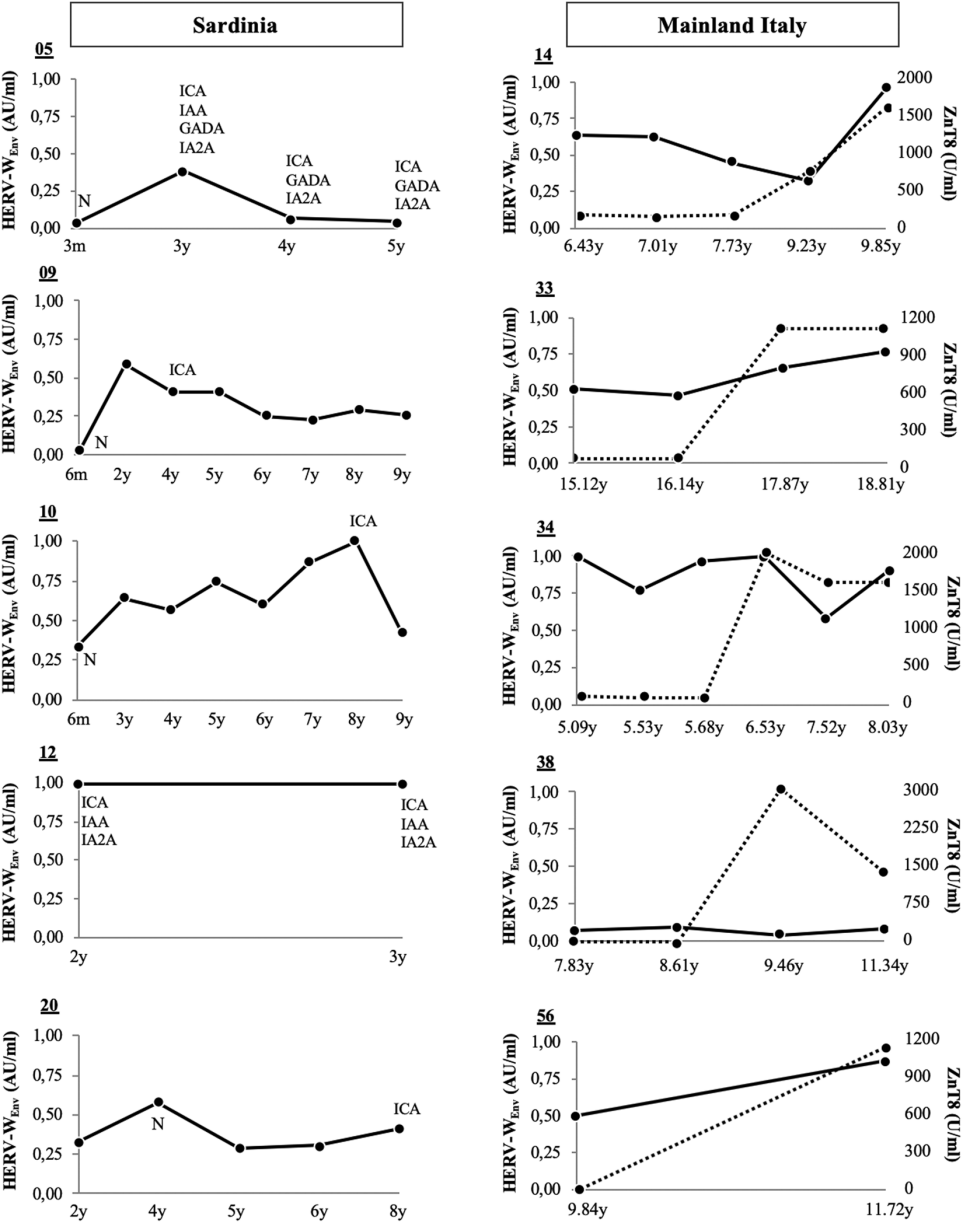
Development of standard Abs in relation to reactivity against HERV-W_Env_ in Sardinian children (**A**) and youths from mainland Italy (**B**) at T1D risk. Patients’ identifiers are reported above the Y axis of each graph. Black line: anti-HERV-W_Env_ Abs values. Dotted line: standard Abs against ZnT8. For Sardinian cohort, the type of diagnostic Abs is specified when positivity threshold has been exceeded. N: measurement not performed. m: months. y: years.

Among Italian subjects at T1D risk who over a period of 4 years developed high titers of standard Abs (n = 5), especially those specific for ZnT8 C-terminal region (>1000 U/ml), 80% showed a strong seroreactivity against HERV-W_Env_ and the homologous peptides (Fig. 3). This biomarker was not included for diagnostic purposes in the other study populations, however, in the Sardinian cohort, increased values registered for HERV-W_Env_ overlaped with or preceded the moment when diagnostic autoantibodies (ICA, IAA, IA2A and/or GADA) were detected.

## Discussion

In this prospective study, we investigated the production of Abs against two agents, namely HERV-W and MAP, in young populations at T1D risk from distinct geographical regions of Italy based on data collected over a 10-year period. Our results indicate a possible role of MAP in HERV-W_Env_ activation which correlates with follow-up levels of diagnostic biomarkers. In addition, the Abs status was evaluated in environmentally unrelated youths with clinical T1D or non-diabetic obesity in order to assess whether developed autoimmunity and risk for metabolic syndrome may determine characteristic serological features.

MAP infection is common only to some exposed subjects and hardly identifiable due to latency phases, extremely low growth and tropism to intestinal macrophages^19^. Although the most severe consequences of MAP proliferation occur in ruminants, the mycobacterium may be easily transmitted to humans with a wide range of contaminated animal products, while anti-MAP Abs have been detected in milk of breastfeeding mothers^20,21^. On the other hand, HERVs are intrinsic sequences disseminated throughout human genome that under certain circumstances may be activated from their silenced form and expressed generating a series of antigens primarily on monocytes and B cells^22,23^. A few studies performed to evaluate the association between HERVs and MAP in autoimmune diseases reported a partial decrease of anti-MAP Abs in multiple sclerosis (MS) following natalizumab treatment which efficiently reduced levels of Abs targeting HERV-W_Env_^24^. No significant difference in specific humoral responses against MAP antigen was obtained when comparing rheumatoid arthritis (RA) patients under treatment with healthy controls^25^. Therapies employed to alleviate MS and RA symptoms act on the immune system, thereby influencing inflammatory responses in general. Outcomes of the present study are based on subjects free from therapy or in the course of insulin treatment, which however does not suppress the reactivity of immune cells. These differences may explain distinct results among the three autoimmune conditions and be the first step to suggest MAP infection as a plausible circumstance able to induce the expression of HERV-W envelope antigens which may result in a further interplay between mycobacterial, viral and human Abs leading in turn to immune imbalance. A possibility of such cascade of events to occur and its link with the development of autoimmunity requires further investigation.

Elevated reactivity and strong correlations between HERV-W_Env_ and MAP/human homologous peptides registered for rT1D youths are in line with this hypothesis. When Abs levels were distributed in more homogeneous profiles over time, values specific for the selected peptides tended to maintain similar trends. In subjects displaying high variations of responsiveness relative to MAP/human antigens, fluctuations specific for HERV-W_Env_ were particularly marked with peak values corresponding to decreasing levels of anti-MAP/human Abs and the lowest values correlating negatively. These shifts may be indicative of sequentiality characteristic for distinct epitopes resulting from a selective responsiveness; the crucial point is to understand whether MAP drives the immunity as a primary antigen. Interestingly, anti-HERV-W_Env_ Abs in T1D youths under insulin therapy equaled to a lower prevalence compared to age-matched rT1D. These subjects also showed significantly increased levels of Abs targeting HERV-K_Env_ even though seropositivity accounted for only 12.16% of the assessed population (Fig. 1C). Past studies based on the viral IDDMK^1,2^ 22 sequence expression excluded the association between HERV-K and T1D^7,9,26^ after an initial report in favor of such a link^27^. Different loci encoding HERV genes may be involved in a potential immune reactivity^28^, nonetheless the absence of significant responsiveness in rT1D would not support a causal role of HERV-K family. In contrast, raised levels of Abs against other two HERV-W epitopes (HERV-W_Env93–108_ and HERV-W_Env140–160_) in diabetic and rT1D youths and a marked degree of correlation with MAP/PI peptides were observed in a pilot cohort (data not shown).

It has been recently reported that MAP infection in cattle affects the expression of immune regulatory genes, including Th17-derived cytokines, interferon regulatory factors, and calcium signaling-associated genes^29^. Both immune activation and inflammation can impair HERV transcription through interaction with specific binding sites in relative HERV promoter regions^30^. Similarly, oxidative stress also seems to contribute by hampering binding of methyl groups transferred by DNA methyltransferases and enhancing gene expression upon inhibition of histone deacytelases^30^. Increased activity of selenium-dependent glutathione peroxidase (GPx) indicative of oxidative processes has been reported in MAP-infected cattle and patients with Crohn’s disease^31^. Formerly, destruction of the exocrine pancreas was observed in a goat with confirmed MAP infection^32^. Recent studies in transgenic mice demonstrated that artificially induced expression of HERV-W_Env_ promotes hyperglycemia, decreased insulin production and immune cell infiltrates in the exocrine part^10^. The hypothesis of MAP as a plausible natural trigger of HERV-W_Env_ transactivation is supported also by the fact that macrophages activated in granulomatous lesions of infected goats highly express CD68^+^ indicative of elevated numbers of lysosomes and acid phosphatase activity^33^. A study in wild ruminants affected by Johne’s disease proved the presence of MAP and cell damage in the pancreatic lymph nodes^34^. Levet *et al*., 2017, reported increased CD68^+^ macrophage infiltrates in T1D exocrine pancreas correlated with HERV-W_Env_ expression^10^. In line with our earlier results, involvement of MAP in the pathogenesis of T1D may occur through a more complex network of interaction that previously thought.

As humans are not the primary target of MAP, it may act indirectly on immune homeostasis as a consequence of survival mechanisms eluding host defense against pathogens and conditions favoring the expression of HERV-W antigens.

Limitations of the study. In this pilot study, we investigated for the first time the interaction between MAP and HERV-W related to T1D, however some limitations couldn’t be avoided. The results were obtained based on three independent populations differing by mean age and the advancement of autoimmunity, each of them forming groups with relatively low numbers of participants. In particular, statistical significance was not attained for a single-point analysis of Sardinian rT1D population compared with age-matched healthy controls. The lack of follow-up samples until development of diabetes in Italian rT1D group and before manifestation of symptoms in Polish T1D population did not allow for a continuous determination of Abs status against the assessed epitopes. Prospective screening involving groups increased in the number of participants is therefore necessary to further assess Abs patterns and a possible transactivation of HERV-W following mycobacterial infection. Investigation of cell-mediated responses and application of *in vivo* models alongside the analysis of retroviral gene expression should be done in order to identify the most probable sequence of events which take place in nature. As well, development of serological markers in numerically extended cohorts of newborns and young children with regard to a possible vertical transmission of Abs needs further evaluation.

## Materials and Methods

### Subjects

Independent cohorts of rT1D subjects were enrolled in two separated centers: 54 children and youths (mean age 9.42 ± 3.84 years, 1:1 male/female ratio) attending the Tor Vergata University Hospital of Rome, Italy, and 14 children (1:2 male/female ratio, mean age 5.85 ± 2.19 years) attending the Department of Diabetes, St. Michele Hospital of Cagliari, Italy. Venous whole blood was collected for the purpose of prospective studies providing in total 103 and 87 follow-up samples for the respective groups over a 10-year period. T1D risk was verified by the presence of disease familiarity with first-degree relatives (parents or siblings), high risk HLA genotype and/or the presence of standard autoantibodies (ZnT8, GADA, IA2A, ICA and IAA). Healthy volunteers (HC) without known history of autoimmune disorders and recent inflammatory episodes were recruited as reference controls following periodic medical checks in the corresponding geographic areas (n = 42, mean age 6.94 ± 3.58 years, 1:1.5 male/female ratio, the Tor Vergata University Hospital of Rome; and n = 17, mean age 6.66 ± 2.59 years, 1:2.4 male/female ratio, Department of Endocrinology, University Hospital of Sassari, Italy). Additionally, a group composed of children/youths with established T1D under insulin therapy (n = 74, mean age 12.9 ± 3.51; females n = 47, males n = 27) and age-matched obese patients (OB; n = 26, mean age 12.54 ± 2.99, mean BMI 27.35 ± 4.53; females n = 16, males n = 10) was enrolled at the Clinic of Endocrinology and Diabetology, Children’s Memorial Health Institute in Warsaw, Poland, for single time-point sample collections in a distinct biogeographic background. General inflammatory background of obesity in an apparent healthy condition often affecting young populations nowadays was considered a valuable control. T1D onset was diagnosed based on the levels of standard biomarkers and levels of glycated hemoglobin, according to the American Diabetes Association criteria^35^. The study protocols were approved by delegated Bioethics Committees (Children’s Memorial Health Institute, Tor Vergata University Hospital, St. Michele Hospital and University Hospital of Sassari) and written informed consent from a parent or legal tutor was obtained for all study participants. All methods were performed in accordance with regional and national regulations.

### Diagnostic autoantibodies

Levels of Abs specific to the ZnT8 C-terminal region (268–369, 325R or 325W) were determined in the sera by Protein A-radioimmunoprecipitation assays according to the protocol by Lampasona *et al*.^36^ with positivity threshold set at 30 U/mL. Abs to insulin, GAD65, and IA-2 were measured by radioligand assays using commercial kits (CentAK® IAA RT, CentAK® anti-GAD65, and CentAK® anti-IA2, Medipan, Germany) according to the manufacturer’s instruction. Values are expressed in arbitrary units with the respective Abs thresholds of >0.4, >0.9 and >0.75 U/mL.

### Antigens

HERV-K_Env19-37_ (SVWVPGPTDDRCPAKPEEE) fragment of envelope surface glycoprotein were selected based on significantly increased seroreactivity in MS and ALS patients observed by Arru *et al*.^37^. Other studies reported comparable responsiveness to a range of HERV-W envelope surface peptides^38^, therefore HERV-W_Env109–123_ (FTQTGMSDGGGVQDQ) was used in the present assessment as a first-trial choice for a pilot correlation analysis. HERV_Env_ peptides along with homologous epitope pairs derived from MAP and human proteins ZnT8 and PI, were synthesized as formerly described^17,37^.

### Serological assays and data analysis

The presence of Abs against the selected antigen set was assessed in separated serum samples through indirect enzyme-linked immunosorbent assay following in-house optimized protocols^14^. Data were normalized to a strongly positive control serum included in all assays with Abs reactivity set at 1.0 arbitrary units AU/mL. Optimal thresholds to discriminate between positive and negative mean values were identified based on the receiver operating characteristic (ROC) curves, setting specificity at 90%. GraphPad Prism ver. 6.0 was employed to determine levels of statistical significance through Mann-Whitney *U* test. Normality of the distribution was assessed by D’Agostino-Pearson test. Comparison of positivity between patients and reference controls was performed using Fisher’s exact test.
